# Deriving a continuum score for group 3 and 4 medulloblastoma tumor samples analyzed via RNA-sequencing or DNA methylation microarray

**DOI:** 10.1016/j.xpro.2023.102509

**Published:** 2023-08-11

**Authors:** James P.R. Hacking, Dean Thompson, Edward C. Schwalbe, Steven C. Clifford, Daniel Williamson

**Affiliations:** 1 Wolfson Childhood Cancer Research Centre, Translational and Clinical Research Institute, Newcastle University for Cancer, Newcastle University, Newcastle upon Tyne, UK; 2 Department of Applied Sciences, Northumbria University, Newcastle upon Tyne, UK

**Keywords:** Bioinformatics, Sequence analysis, Cancer, Sequencing, RNAseq, Molecular Biology

## Abstract

Here, we present a protocol for deriving a continuum score for group 3 and 4 medulloblastoma tumor samples analyzed via RNA-sequencing or DNA methylation microarray. We describe steps for utilizing NMF-defined group 3/group 4 metagenes to calculate a continuum score between 0 and 1 that can be projected onto new sample data analyzed via RNA-sequencing. We then detail procedures for reverse engineering a continuum score for samples analyzed via DNA methylation microarray using a random forest classifier.

## Highlights

• A protocol to generate medulloblastoma group 3/group 4 continuum scores
• Input either expression or methylation data
• Run samples through R pipeline or through Shiny app
• Generate a score between 1 and 0 to place patients on the continuum

## Abstract (editor highlights)

Publisher’s note: Undertaking any experimental protocol requires adherence to local institutional guidelines for laboratory safety and ethics.

## Graphical abstract

**Figure:**
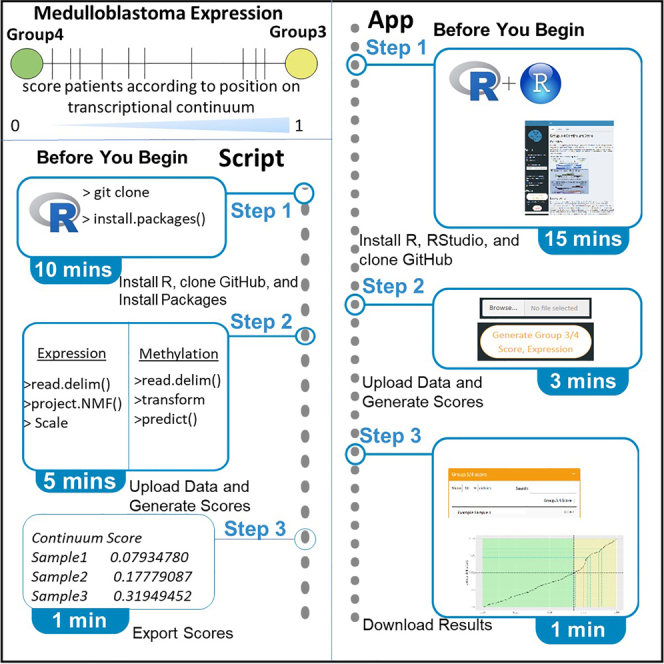

## Before you begin

The protocol below describes the specific *in-silico* steps for deriving a continuum score from group 3/group 4 medulloblastoma sample data analyzed using RNA-sequencing or DNA methylation microarray (either Illumina 450k or EPIC platforms). Determining sample subgroup is therefore an essential first step. This can be determined via the upload of DNA methylation microarray sample data (IDAT format) to the Molecular Neuropathology Classifier website (v11.4 and v12.5 subgroup calls are compatible – link: https://www.molecularneuropathology.org/mnp/). Prior knowledge of R, RStudio and data processing for tumor sample data from RNA-sequencing and DNA methylation microarray sources is also assumed: please refer to the original publication^1^ and key resources table section for detail on platform-specific pre-processing strategies and software used.

To derive a continuum score for group 3/group 4 tumors analyzed via RNA-sequencing, an NMF (Non-negative Matrix Factorization) model trained using 331 medulloblastoma tumors is projected onto incoming sample data using a procedure broadly outlined by Tamayo et al.^2^ This procedure is somewhat resistant to noise, differences in platform or even species. Two of the metagenes which correspond to Group 3 and Group 4 patients are logistically transformed and a ratio between the two is produced that is then scaled between 0 and 1 (G3/G4 score). Whilst knowledge of metagenes and NMF is not expressly required for this protocol, more information about NMF and how it is applied can be found in the original publication.^1^

By utilizing paired expression-methylation profiles where RNA-based continuum score was known (n = 192/331), a random forest regression model was trained using 115 CpGs. This enables reverse engineering of a continuum score for group 3/group 4 samples, with high accuracy (RMSE = 0.036) and without the explicit need for an expression profile i.e., from either an Illumina Methylation450k or MethylationEPIC profile alone. More detail on how the random forest model classifier was trained is provided in the original publication.^1^

### Hardware preparation

Each step of the protocol has been tested within R Studio using R version 4.2.2. Both command line and shiny app tutorials can be executed using a standard work machine with 16-GB RAM and has been tested under Linux (running Ubuntu 20.04.5 LTS), MacOS and Windows operating systems.

## Key resources table

**Table:** 

REAGENT or RESOURCE	SOURCE	IDENTIFIER
**Deposited data**		

NMF Model (*nmf.res*)	Williamson et al.^1^	https://github.com/hackingjpr/Group3-4App/blob/main/StarProtocols_Guide/data/nmf.res.rds
Random Forest Classifier (g3.g4.cont.rfe.Rdata)	Williamson et al.^1^	https://github.com/hackingjpr/Group3-4App/blob/main/StarProtocols_Guide/data/g3.g4.cont.rfe.Rdata
Test Expression Dataset (tpms.mat)		https://github.com/hackingjpr/Group3-4App/blob/main/StarProtocols_Guide/data/tpms.mat.txt
Test Methylation Dataset (mvals.mat)	Schwalbe et al.^3^	https://github.com/hackingjpr/Group3-4App/blob/main/StarProtocols_Guide/data/mvals.mat.txt

**Software and algorithms**		

R v4.2.2		https://www.r-project.org
Bioconductor		https://bioconductor.org
MASS_7.3-51.6	Venables and Ripley^4^	https://www.stats.ox.ac.uk/pub/MASS4/ .
NMF_0.24.0	Gaujoux and Seoighe^5^	https://cran.r-project.org/web/packages/NMF/index.html
Biobase_2.50.0	Huber et al.^6^	https://www.bioconductor.org/packages/release/bioc/html/Biobase.html
BiocGenerics_0.36.1	Huber et al.^6^	https://www.bioconductor.org/packages/release/bioc/html/BiocGenerics.html
cluster_2.1.1		https://cran.r-project.org/web/packages/cluster/index.html
rngtools_1.5.2		https://cran.rstudio.com/web/packages/rngtools/index.html
pkgmaker_0.32.2		https://cran.rstudio.com/web/packages/pkgmaker/index.html
registry_0.5-1		https://cran.r-project.org/web/packages/registry/index.html
randomForest_4.6-14	Liaw and Wiener^7^	https://cran.r-project.org/web/packages/randomForest/index.html
caret_6.0-86	Kuhn^8^	https://cran.r-project.org/web/packages/caret/index.html
mlbench_2.1-1		https://cran.r-project.org/web/packages/mlbench/index.html
lattice_0.20-45		https://cran.r-project.org/web/packages/lattice/index.html
bumphunter_1.32.0	Jaffe et al.^9^	https://www.bioconductor.org/packages/release/bioc/html/bumphunter.html
minfi_1.36.0	Aryee et al.^10^	https://www.bioconductor.org/packages/release/bioc/html/minfi.html
shiny_1.7.2		https://cran.r-project.org/web/packages/shiny/index.html
DT_0.23		https://cran.r-project.org/web/packages/DT/index.html
ShinyWidgets_0.7.2		https://cran.r-project.org/web/packages/shinyWidgets/index.html
Shinydashboard_0.7.2		https://CRAN.R-project.org/package=shinydashboard/index.html
Ggplot2_3.3.6		https://ggplot2.tidyverse.org
Ggpubr_0.4.0		https://CRAN.R-project.org/package=ggpubr/index.html
Foreach_1.5.2		https://CRAN.R-project.org/package=foreach/index.html
Waiter_0.2.5		https://CRAN.R-project.org/package=waiter/index.html
Caret_6.0-92	Kuhn^8^	https://CRAN.R-project.org/package=caret/index.html
NMF_0.24.0	Gaujoux and Seoighe^5^	https://cran.r-project.org/web/packages/NMF/index.html
RandomForest_4.6-14	Liaw and Wiener^7^	https://cran.r-project.org/web/packages/randomForest/index.html
BiomaRt_2.46.3	Durinck et al.^11^	https://bioconductor.org/packages/release/bioc/html/biomaRt.html
GridExtra_2.3		https://CRAN.R-project.org/package=gridExtra/index.html
Survival_3.2-7	Therneau^12^	https://CRAN.R-project.org/package=survival/index.html

**Other**		

GitHub repository		https://github.com/hackingjpr/Group3-4App.git https://doi.org/10.5281/zenodo.7948042

## Step-by-step method details

### Continuum score assignment (RNA-sequencing)

**Timing: ∼5–15 min (Timing can vary depending on the number of samples in your dataset)**

Here, we project our existing NMF model onto a previously unseen group 3/group 4 medulloblastoma RNA-sequencing dataset to derive a continuum score for each sample. Several R packages, custom functions and data objects are required. Data objects and functions can be found in the associated GitHub repository (https://github.com/hackingjpr/Group3-4App). We also provide sample usage data along with expected outputs in the GitHub. All *in-silico* analyses detailed in this section are performed in R and require the following lines of code to be run to set up the package environment and load the necessary data objects prior to continuum score assignment:

Within terminal.

1. Clone GitHub repository.

> git clonehttps://github.com/hackingjpr/Group3-4App.git/your/directory/

*# This will download the git repository into a directory of your choosing*

Within RStudio.

2. Install/load required R packages and their dependencies.

> install.packages("NMF", dependencies = TRUE)

> install.packages("MASS", dependencies = TRUE)

> BiocManager::install("biomaRt")

*# For specific package versions, see*key resources table*section. When confronted with*

*yes/no questions, answer yes to install dependency packages.*

> library(NMF)

> library(MASS)

*# This loads the packages required into your working environment.*

3. Load required data objects.

**CRITICAL:** You MUST update ‘/your/directory/’ to the location which you cloned the GitHub repository in step 1.

> nmf.res <- readRDS(file = "/your/directory/Group3-

4App/StarProtocols_Guide/data/nmf.res.rds")

*# This loads in the precalculated NMF model.*

4. Load the required custom functions.

> source(file = "/your/directory/Group3-

4App/StarProtocols_Guide/R/Project_NMF.R")

#*Wrapper function used to project NMF model onto unseen group3/group4 sample data. A function breakdown is provided below (see*Figure 1*)*

5. Load sample data as a matrix object.

**Figure 1:**
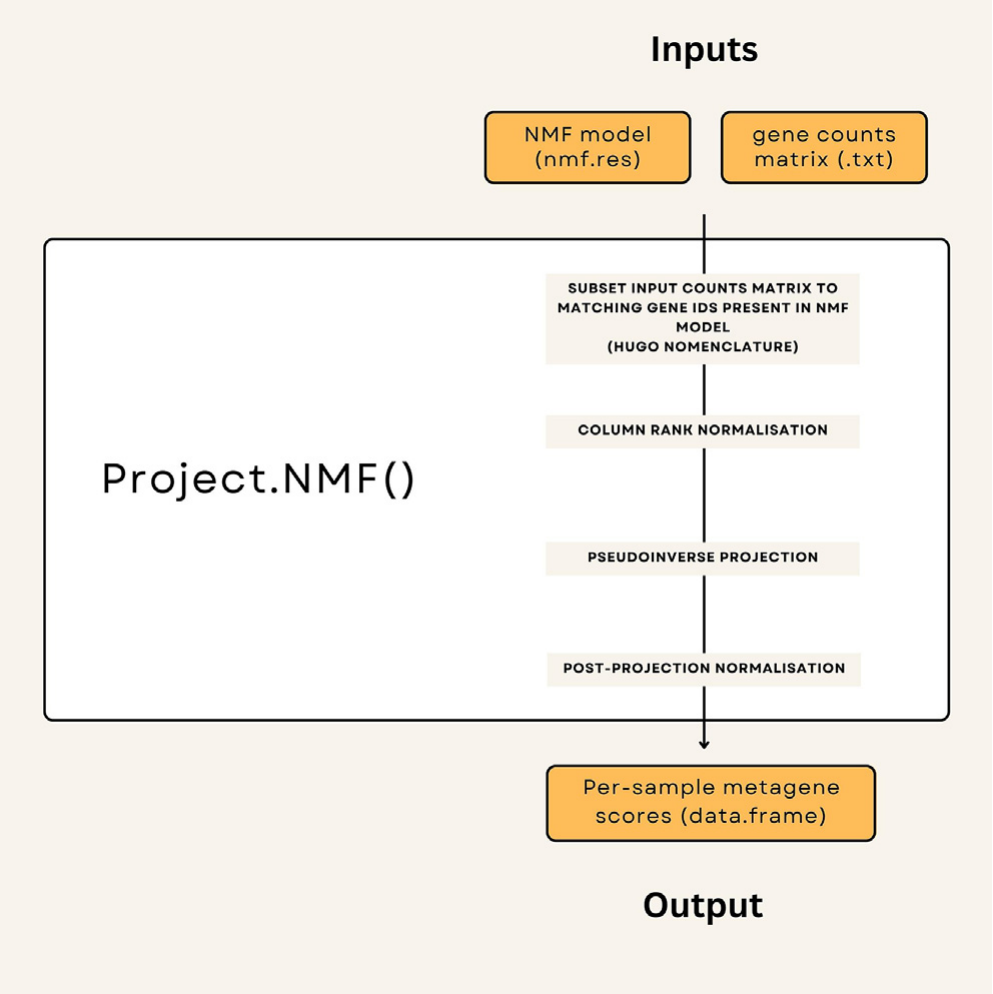
Step-by-step breakdown of project.NMF() function

> tpms.mat <- read.delim("/your/directory/Group3-

4App/StarProtocols_Guide/data/tpms.mat.txt")

**CRITICAL:** If you wish to use your own RNA-sequencing sample data, you must ensure that it follows the same format as tpms.mat. This object is a matrix, where columns correspond to samples and rows correspond to genes, with expression counts presented in the transcripts per million (TPM) format or equivalent. All genes (rows) must use HUGO gene nomenclature i.e., gene symbols. If your input dataset is not annotated correctly, please see problem 1 in the troubleshooting section. Note that a column-rank normalization procedure is employed, this coupled with the NMF projection and other normalization procedures renders the results somewhat resistant to noise and compatible with representations of expression other than TPM. We have for example used Rlog, or variance stabilized transforms from DESeq or even other platforms such as Affymetrix microarray or nanostring data with success.

***Note:*** When projecting onto platforms other than bulk RNA-seq, appropriate filtering strategies to remove invariant genes/probes may be necessary.

6. Project NMF model onto sequencing data

> tpms.H <- project.NMF(input.array = as.matrix(tpms.mat),

nmf.result = nmf.res)

*#Apply project.NMF function to input dataset. Function breakdown is provided below (see*

Figure 1*)*

7. Extract Group 3 and Group 4 metagenes from data and transpose matrix.

> g3g4.tpms <- t(tpms.H[c(3,1),])

*#Rows 3 and 1 in tpms.H correspond to the metagenes for Groups 4 and 3 respectively*

8. Apply logistic transformation to metagenes.

> logistic.g3g4.tpms <- apply(g3g4.tpms,2,function(x){(1 / (1 + exp(-x)))})

*# apply a logistic transformation*

> logistic.g3g4.tpms.score <-apply(logistic.g3g4.tpms,1,function(x){x[2]/(x[1]+x[2])})

*# calculate a ratio between logistically transformed Group3 and Group4 metagene*

9. Scale values between 0 and 1.

> scaling.function <- function(x){(x-min(x)) / (max(x)-min(x))}

*# create a function to scale values between 0 and 1*

> logistic.g3g4.tpms.continuum.score <- scaling.function(logistic.g3g4.tpms.score)

*# apply the function to the unscaled g3g4 scores*

**CRITICAL:** If you are using a small dataset or one that does not represent the full spectrum of Group 3/Group 4 medulloblastomas you may want to omit this step and present unscaled G3/G4 ratios (see Limitations below).

***Alternatives:*** You may wish to append to the precalculated G3/G4 ratios from Williamson et al. and then scale together with your new samples in which case the following alternative command should be used:

> scaling.function1 <- function(x){(x - 0.3953062) / (0.5964371

- 0.3953062)}

*# create a function to scale values between 0 and 1 using Williamson et al. data)*

> logistic.g3g4.tpms.continuum.score <-

scaling.function1(logistic.g3g4.tpms.score)

*# Apply scaling*

***Note:*** Theoretically, this could lead to some sample returning values under 0 or over 1. The user would need to take a considered view on such samples. They could simply be producing values close to 1 or 0 but otherwise consistent with samples at the extreme limits of the G3/G4 continuum, in which case manually assigning them the maximum 1 or minimum 0 value may be a valid approach. Should they massively exceed previous limits they may simply be outliers or technical artefacts that would be best noted but excluded from further analysis.

10. Present output as data.frame for export.

> logistic.g3g4.tpms.continuum.score <-

as.data.frame(logistic.g3g4.tpms.continuum.score)

> colnames(logistic.g3g4.tpms.continuum.score) <- 'Continuum Score'

*# Renaming for easier interpretation*

> write.csv(logistic.g3g4.tpms.continuum.score, file =

'/your/directory/my_continuum_scores.csv ', row.names = TRUE)

*#Export as .csv table*

### Continuum score assignment (DNA methylation microarray)

**Timing: ∼5–15 min (Timing can vary depending on the number of samples in your dataset)**

Here, we project our existing NMF model onto a previously unseen group 3/group 4 medulloblastoma DNA methylation dataset to derive a continuum score for each sample. Several R packages, custom functions and data objects are required, each of which can be found in the associated GitHub repository (https://github.com/hackingjpr/Group3-4App). We also provide example usage data along with expected outputs in the GitHub. All *in-silico* analyses detailed in this section are performed in R, and require the following code to be run to set up the package environment and load the necessary data objects prior to continuum score assignment:

11. Install/Load required packages and their dependencies

> install.packages('mlbench', dependencies = TRUE)

> install.packages('caret', dependencies = TRUE)

> install.packages('randomForest', dependencies = TRUE)

*# For specific package versions, see*key resources table*section.*

> library(mlbench)

> library(caret)

> library(randomForest)

*# This loads each package into your working environment*

**CRITICAL:** You MUST update ‘/your/directory/’ to the location which you cloned the GitHub repository in step 1 of Continuum score assignment (RNA-Sequencing).

12. Load in the prediction object

> load(file = "/your/directory/Group3-

4App/StarProtocols_Guide/data/g3.g4.cont.rfe.Rdata")

*# This loads in the precalculated random forest model*

13. Load in example methylation dataset.

> mvals.mat <- read.delim("/your/directory/Group3-

4App/StarProtocols_Guide/data/mvals.mat.txt")

**CRITICAL:** The random forest model in this protocol requires that test data be provided as a matrix of M-values (logit-transformed beta values), where columns correspond to sample ID and rows correspond to probes. If your data is a matrix of beta values (object below named as “your.betas”), you can easily convert these to M-values using the following:

> mvals.mat <- log2(your.betas/(1-your.betas))

*#logit-transformation*

14. Subset M-Value matrix to probes used as predictors in model

> mvals.mat <- as.matrix(mvals.mat[predictors(g3.g4.cont.rfe),])

*#Removes probes that are not used for prediction*

***Alternatives:*** If you are working with data obtained using other methylation platforms such as bisulfite sequencing (BS-Seq), it is still possible to derive a continuum score. A typical BS-Seq methylation data output will contain Chromosome, Start and End columns which can be used to ‘lift’ over CpGs that map to those covered by the Illumina Methylation Manifest. This will create a matrix of BS-Seq derived beta values that are annotated with associated Illumina probe IDs that can be used to subset to the required predictors in the random forest model. Whilst BS-Seq methylation values are known to be correlated to those obtained from the methylation array platform and in theory should produce similar predicted continuum scores, it should be noted that the random forest model in this protocol was not explicitly trained using sample data from this platform. An example script that can be used to achieve this is provided in a separate GitHub related to processing whole genome bisulfite sequencing data at https://github.com/tezla93/WGBS_MB/blob/main/InterPlatform/Liftover.R

15. Apply test set to model and get predicted continuum scores using predict()

> pred.cont.rand.for <- as.data.frame(predict(g3.g4.cont.rfe,

t(mvals.mat)))

> write.csv(pred.cont.rand.for, file =

'/your/directory/my_continuum_scores_Methylation.csv', row.names = TRUE)

*#Export as .csv*

### Interactive shiny app

**Timing: ∼5–10 min (Timing can vary depending on number of samples in your dataset)**

An interactive Shiny app has been created in which user’s datasets can be uploaded, continuum scores obtained, and downloaded, in either CSV or PDF format. The relevant scripts and files can be downloaded from the GitHub repository created to accompany this publication, this is located at: https://github.com/hackingjpr/Group3-4App.git, an in-depth tutorial can be found in the GitHub Readme and on the app itself, there is also a tutorial video (Methods video S1). If you wish to run the app on your own system, you may clone the GitHub repository by directly downloading from the repository (see ‘Continuum Score Assignment (RNA-Seq)’ section above.)

Methods video S1. A video tutorial covering steps 1–4 of the Shiny App

You may run the app on your machine using RStudio. Upon opening the “app.R″ file you will either be able to run the app or will need to run the whole block of code so that RStudio can recognize that it encodes a Shiny app. To run a block of code select it all with CTRL+ A and then run it by pressing CTRL+ENTER. Further installation information is included in the Group3-4App (https://github.com/hackingjpr/Group3-4App.git) repository’s ReadMe file (README.md).

16. Select Expression or Methylation and Upload Data: Select the appropriate button on the left-hand side of app and then press “Browse...”

***Note:*** For Methylation idat files, we recommend uploading no more than 10 files at a time; this will speed up the score generation and keep the PC’s memory use low. There is no limit for expression samples, although with too many the resulting plots can begin to look crowded.

**CRITICAL:** If using your own RNA-sequencing sample data, ensure that it follows the same format as tpms.mat. tpms.mat is a matrix, where columns correspond to samples and rows to genes. Expression counts need to be presented in the transcripts per million (TPM) format or equivalent. All genes (rows) must use HUGO gene nomenclature, i.e., gene symbols. If your input dataset differs to this, please see problem 1 in the troubleshooting section.

17. *Generate Group 3–4 Scores* by clicking on the “Generate Group 3/4 Score” button.

*Note:* The app will take you to the results tab once the scores are calculated. This tab will contain a table displaying your samples and their group 3/4 score and three different plots. These plots include placing your samples along the group 3/4 continuum and two survival curves. The first being a general survival curve based on the patient’s score and the second showing the effect that age can have on patient’s survival.

18. *Export Data*: Navigate to the Download tab, which is above your results. You will export the results in either CSV or PDF format. CSV will be the raw table and PDF will contain the table as well as any plots. Figure 2 shows an example of the plots generated in the PDF file.

**Figure 2:**
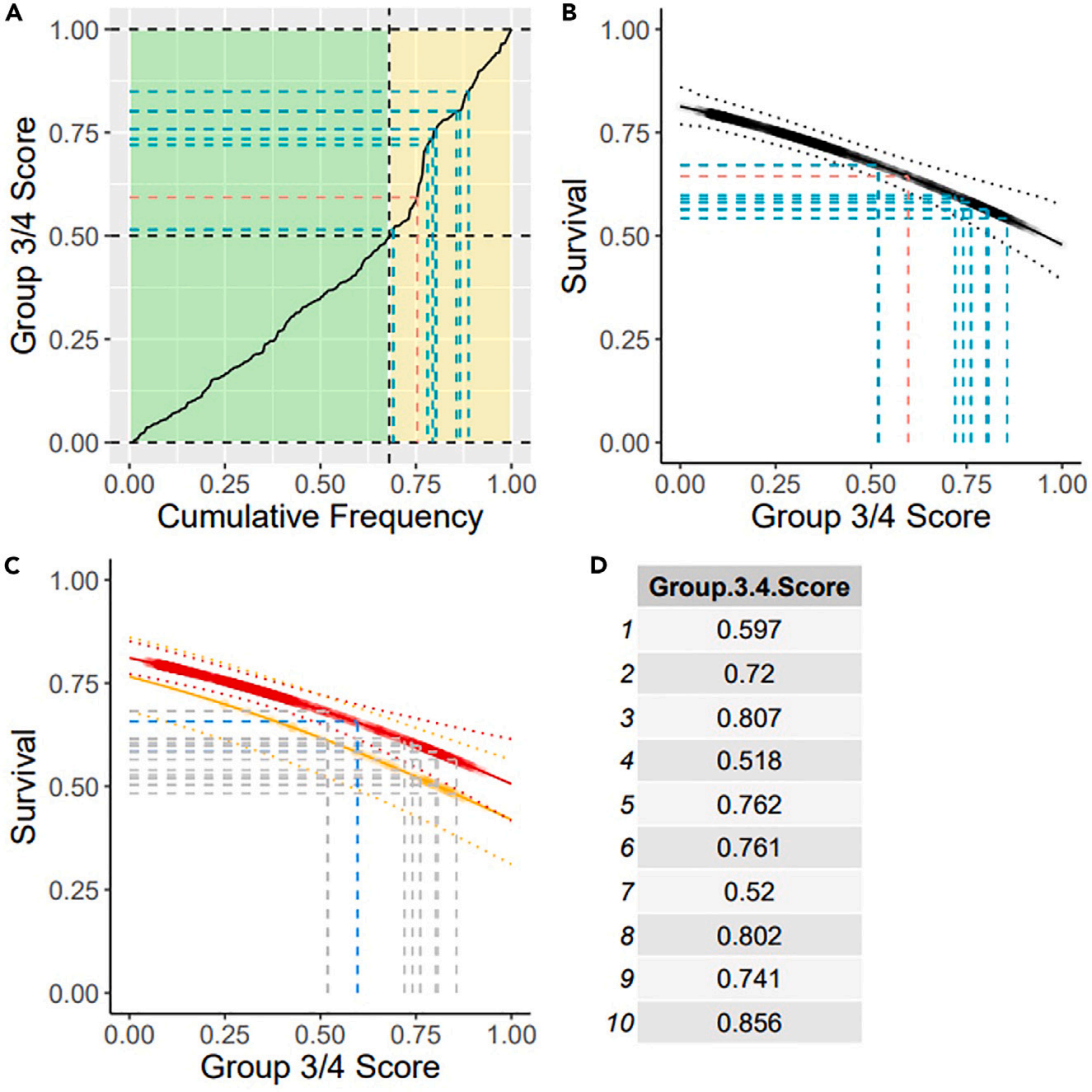
An example of the plots generated and exported to PDF (A) Cumulative frequency plot showing where your samples lie upon the continuum. (B) Survival curve with no risk factors accounted for. (C) Survival curve taking into account patient’s age. (D) Table of raw group 3–4 scores.
19. *Reset*: If more samples are to be tested then press the reset button, this takes you back to the start and allows you to upload more samples.

## Expected outcomes

### Continuum score assignment (RNA-sequencing or DNA methylation microarray)

The primary expected outcome of this protocol is a table presenting a list of group 3/4 continuum scores for each submitted medulloblastoma sample (analyzed via RNA-Sequencing or Methylation Microarray). These tables can be viewed and analyzed further (e.g., plotting or looking for survival associations) within R or saved to be used at a later date.

### Shiny app

Another outcome of this protocol is the ability to calculate group 3/4 continuum scores and generate plots in an interactive shiny app. This app allows users to generate group 3/4 scores without having to have knowledge of coding. The outputs of this app are then exportable for further study. For testing the app, or script, we have uploaded a number of sample files to the GitHub repository, the results generated by these files are in our GitHub repository’s Readme file.

The resulting continuum scores can then be used for a number of applications. Our paper^1^ and others have suggested that the patterns of transcription reflected by the continuum score are highly associated with prognosis, the state of activation of specific biological pathways, clinico-pathological characteristics and the specific point in early development at which tumourigenesis was first initiated. This protocol can be used by researchers wishing to investigate their own expression or methylation data in the future, giving a reference point whereby a common metric can be used to allow meaningful cross-comparison. For example, one could take a fresh collection of patients and calculate their group 3/4 scores allowing you to validate or create your own risk-models, which incorporated G3/G4 score. It could also be used to place individual cells on the continuum by using single cell data giving a measure of both intra-tumoral heterogeneity as well as the average (pseudo-bulk) score for that individual. If building or using models of medulloblastoma - for example genetically engineered mice, PDX or cell lines - a methylation or expression profile could be used to know specifically which part of the transcriptional continuum your model represented.

## Limitations

There are a number of limitations to this protocol which we wish to highlight here. First, this protocol is, of course, only useful for group 3 or 4 medulloblastoma. In future this could be expanded to different cancer types and different data structures. This is outside the scope of this publication but could be an interesting and highly useful future piece of work.

Second, for your results to be valid you must be confident that each of the samples you are analyzing are specifically Group 3/Group 4 medulloblastoma. It is beyond the scope of the paper to outline how this should be done and many other publications have described this.^1^^,^^2^^,^^13^^,^^14^ However, if using a DNA methylation array, it would be sensible to check using the Molecular Neuropathology Classifier website (https://www.molecularneuropathology.org/mnp/). For expression data, an integrated clustering with pre-existing medulloblastoma reference expression sets (e.g., Williamson et al., 2022,^1^ Cavalli et al., 2017^14^) and an examination of critical expression markers may be of use.

Third, analyzing limited numbers of expression profiles can cause issues with scaling of the final G3/G4 score; this issue does not arise with DNA methylation analysis. The protocol outlined above assumes that a sizeable number of other Group 3/Group 4 expression profiles were being analyzed and that they were broadly reflective of the extent of the Group 3/Group 4 continuum as was the case in the original publication.^1^ If analyzing only Group 3 samples, for example, or just a single Group 3/4 subtype (I-VIII). This would not influence the metagene projection *per se* but when it came to scaling the resultant G3/G4 score to between 0 and 1 the resultant score would be skewed. Nevertheless, the relative differences in G3/G4 ratio should be internally consistent within any dataset analyzed and researchers may find it helpful to simply omit the scaling step and produce an unscaled G3/G4 ratio. As an alternative solution we provide the scaling ratios from the cohort analyzed in the paper which can be added to smaller datasets for the purposes of scaling to give an approximation of the G3/G4 score as reported in the Williamson et al. study.^1^

Fourth, despite ensuring that samples are *bona fide* Group 3/Group 4 medulloblastoma and even when using sizeable cohorts, it is possible to obtain outliers which again may skew the scaling; one such case was reported in the Williamson et al. 2022 paper.^1^ In such instances it is wise to exclude these samples. Outliers can be defined as any sample which exceeds ±3 standard deviations from the mean when considering the unscaled G3/G4 ratio but any reasonable definition is valid. The important consideration is to be aware how this may affect your results.

Finally, within the app we present for the purposes of illustration some of the Cox regression models (survival analysis) and molecular pathology presented within the Williamson et al. 2022 study.^1^ This is so your samples can be placed within the context of those historical and retrospective cohorts. In no way should this be deemed to be making any kind of usable clinical prediction about an individual’s risk of death or clinico-pathology outside of the extremely limited sphere of academic research. This is provided purely for research purposes and disclaimers within the app make this clear.

## Troubleshooting

### Problem 1

When projecting my input RNA-Seq dataset onto the NMF model, I get the following error message.

**Figure:**
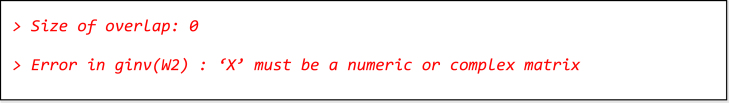

**Likely cause:** RNA-Seq dataset has not been annotated with HUGO gene IDs.

### Potential solution

Annotate your dataset with HUGO gene IDs using biomaRt.

Step-by-step:

• Install biomaRt

> BiocManager::install("biomaRt")

> library(biomaRt)

• Isolate the unique identifiers for each transcript in your dataset (e.g., Ensembl gene IDs) - Here, we have provided an example dataset (problem1.tpms) that is incorrectly annotated with Ensembl gene identifiers to demonstrate how this can be corrected.

> problem1.tpms <- read.delim(“/directory/to/Group3-

4App/StarProtocols_Guide/data/problem1.tpms.txt”)

> ensembl_IDs <- rownames(problem1.tpms)

• Create a biomaRt connection to database for *Homo Sapiens*

> mart<- useMart(biomart = ‘ensembl’,

   dataset = ‘hsapiens_gene_ensembl’)

• Annotate each Ensembl identifier with its respective gene symbol:

*#This deals with an object labelled with hgnc symbols – If you are*

*not using hgnc_symbols, change this accordingly.*

> symbols <- getBM(attributes = c(‘ensembl_gene_id’, ‘hgnc_symbol’),

     filters = ‘ensembl_gene_id’,

     ensembl_IDs,

     mart = mart)

• Replace your current identifiers with gene symbols:

> annotatedix <- match(ensembl_IDs, symbols$ensembl_gene_id)

> symbols[annotatedix,] -> annotatedGenes

> problem1.tpms$hgnc_symbol <- annotatedGenes$hgnc_symbol

#Remove duplicated rows using:

> problem1.tpms <- problem1.tpms [!duplicated(problem1.tpms

$hgnc_symbol),]

#Remove transcripts with no gene annotation using:

> problem1.tpms <- na.omit(problem1.tpms)

#Set gene IDs as rownames and remove column:

> rownames(problem1.tpms) <- problem1.tpms$hgnc_symbol

> problem1.tpms$hgnc_symbol <- NULL

### Problem 2

The app will not run on first deployment.

**Likely cause:** If it is the first time loading up the app on your own machine it is likely the issue is due to packages wanting to be updated. The first time you run the app it has to install a number of packages that all have sub dependencies. Also. whilst it is true that the app automatically installs the relevant packages at the time of testing and running on the machines we had available, it is possible that certain CRAN/Bioconductor packages and their dependencies get updated over time in a way that prevents the packages from installing automatically on your machine.

### Potential solution

In the bottom left quadrant in RStudio there should be a question along the lines of “Update (Y/N)”. Typing Y and then pressing Return will lead to R updating the necessary packages. Also pay attention to the console in RStudio if it indicates an error installing a specific package then you may need to manually install certain packages e.g., running install.packages(“my_package”) command for CRAN packages.

### Problem 3

The app will not open after an error caused it to close.

**Likely cause:** Efforts have been made to minimize errors that lead to the app closing itself but of course it may still happen in some situations. If it does then RStudio may still be running the script, even though you cannot see the app.

### Potential solution

In the top right corner of the bottom left quadrant box there should be a small red octagon with “STOP” written on it. If you click this the script will stop running and you can re-open the app.

## Resource availability

### Lead contact

Further information and requests for resources and reagents should be directed to and will be fulfilled by the lead contact, Dan Williamson (email: daniel.williamson@newcastle.ac.uk).

### Materials availability

All materials required to complete this protocol are available at https://github.com/hackingjpr/Group3-4App.

### Data and code availability

All data and code required to complete this protocol are available at https://github.com/hackingjpr/Group3-4App.
